# The multivariate approach identifies relationships between pre-slaughter factors, body lesions, ham defects and carcass traits in pigs

**DOI:** 10.1371/journal.pone.0251855

**Published:** 2021-05-20

**Authors:** Marika Vitali, Paolo Bosi, Elena Santacroce, Paolo Trevisi

**Affiliations:** Department of Agricultural and Food Sciences and Technologies (DISTAL), University of Bologna, Bologna, Italy; INIA, SPAIN

## Abstract

Abattoir meat inspection has been proposed for the collection of welfare outcomes. The identification of suitable animal-based measures (ABM) is still a critical point that needs to be implemented to avoid collinearity among measures. The present study aims to benchmark the presence of ABM such as skin and tail lesions and ham defects in carcasses from 79 batches of Italian Heavy pigs and to identify possible relationships between the assessed ABM and pre-slaughter factors such as the season and the overnight lairage. Furthermore, the study also considers the effect of pre-slaughter conditions and ABM on carcass traits parameters (cold carcass weight and lean meat percentage). Skin and tail lesions were recorded at the slaughter line. The presence of abscesses, muscle tears and veining defects were assessed in the hams at trimming, according to the Parma Ham Consortium. A multivariate analysis was performed to identify relationships between ABM and pre-slaughter factor; therefore, a linear model was built to assess the effect on carcass weight and lean meat percentage. Main welfare issues were represented by skin and tail lesions and muscle tears (prevalence above 10%). Multivariate analysis evidenced that skin lesions and veining defects were mostly associated with the warm season. Abscesses and muscle tears in the hams were more likely related to overnight lairage, while tail lesions contributed equally to both season and lairage. Moreover, lairage related factors showed to affect lean meat percentage. The findings of the present study suggest that ham defects might be useful indicators of pre-slaughter stress. The validation of these findings with physiological parameters could be of interest for further studies.

## Introduction

Abattoir meat inspection, besides the main role on the safeguard of meat safety [1] has been proposed for the collection of animal-based welfare outcomes [2]. Recoding data at abattoir can provide substantial information to qualify the standard of animal housing and management during the rearing period and/or pre-slaughter [3–5]. Recently, the European Food Safety Authority published a report that highlights the usefulness of animal-based measures to assess the welfare of pigs at slaughter [6]. This report evidences the need to identify more indicators and to validate them, exploring the complex relationship between risk factors and animal-based measures (ABM). Recording these indicators after slaughter can provide a retrospective method to identify hazards during the marketing chain and to identify the association between pre-slaughter factors and meat quality. One of the most reliable ABM is the presence of skin and tail lesions on the carcass [7]. Skin lesions can occur during all the phases of rearing, however, those inflicted in the last 48 hours of life are considered the most frequently observed at slaughter and impacting the meat quality [8, 9]. In the pre-slaughter period, skin lesions can originate from different sources, such as overcrowding at loading or during transport, fighting after regrouping at lairage, mounting due to hierarchy or bruises following a blunt trauma [8, 10, 11]. Skin lesions represent an economical issue resulting in reduced profits due to carcass downgrading [12], especially in typical productions, where skin integrity is fundamental for the seasoning procedure, as in the Parma Ham PDO production [13]. Tail lesions are the outcome of tail biting behaviour in pigs. Tail biting is known as one of the main challenges in pig production since its occurrence is considered as an iceberg indicator of multifactorial welfare problems in pig herds [14]. For this reason, the tail lesion could be an unspecific indicator of poor welfare, and its importance as ABM for the assessment of the welfare pre-slaughter is uncertain [6]. Despite that, tail lesions have been considered also an important cause of economic loss for the industry since it has been associated with a reduction of productive performances, an increased susceptibility to diseases and infections, as well as poor carcass traits [12]. Tail biting can arise during all phases of pig production, and it can occur once or be reiterated in time [15, 16]. It is a source of stress, pain, and health impairment for the victim, as well as a symptom of psychological stress in the biter [15]. Therefore, it is important to consider this type of lesion when assessing animal welfare at any stage.

When considering new ABM, one possibility is the evaluation of hams after they have been portioned from the carcass. In heavy pig production, this can be done the day after slaughter when hams are selected for the product of designed origin (PDO). According to the Parma Ham Consortium, some called “defects” are clinical signs, such as haematomas, petechial haemorrhaging, abscesses and muscle tears. These conditions are responsible for the downgrading of the ham from the PDO circuit, with sensitive loss of profit for the farmer (estimated to -30% of the value [17]) which can be attributable to pre-slaughter conditions and/or infectious agents. Muscle tear and haematomas might be caused by unloading or be a consequence of fighting during lairage, or poor handling [6]. The abscesses can have a traumatic or infectious origin, and their development might be favoured by many conditions, for instance, the host immunity and physiology, stress or other pathologies. PSE- and DFD- like defects might depend on pre-slaughter stress [18] or to be artefacts due to improper carcass handling (i.e. excessive water temperature or singing).

Veining defect is, on the contrary, not attributable to previous pathologies and it has not a well-identified aetiology. It can be described as the presence of evident subcutaneous veins, observed a few hours after slaughter [19]. These hams are excluded from the PDO circuit because it affects the visual aspect of hams [19]. Nowadays there is no certainty about risk factors for veining defects, even if some risk factors have been hypothesized, such as globosity of the ham, leanness, type of stunning, season and lairage lasting [17, 19, 20]; however, the results obtained do not seem very conclusive.

The present study aimed to benchmark the presence of ABM in batches of Italian Heavy pigs. Then the study aimed to identify the relationships between the assessed ABM and pre-slaughter factors. Lastly, the study also considered the effect of pre-slaughter conditions and ABM on carcass traits parameters.

## Materials and methods

### Ethics approval

The observations involved batches of pig carcasses randomly selected after slaughter, and no pigs were killed specifically for the study. Moreover, before the experiment began, the procedure was explained to the slaughterhouse staff. No tissue or any other sample was collected, therefore according to the EU legislation DL n. 26, 24/03/2014 there was no need for the approval of the experimental protocol by the Italian Health Ministry.

### Experimental protocol

#### Batch of pigs

Data collection was carried out during 11 days from June to December 2019 in a single slaughterhouse placed in northern Italy and characterised by a weekly throughput of approximately 12,000 pigs. Slaughter was performed by exsanguination in the prone position, after head-only electrical stunning, and the speed of the dressing line was 6 seconds between one carcass and another. A total of 79 batches, randomly selected, were recorded from 43 farms, for a total of 10,079 carcasses observed. All of the farms were part of the PDO Parma Ham Consortium [21], according to which the pigs must be slaughtered at a live weight of 160 kg ±10% of live body weight and at least 9 months of age.

#### Pre-slaughter conditions

For each batch, lairage lasting and season of slaughter were recorded. For each batch, the seasons of slaughter was assigned according to the following categories: warm (W) = from June to September; cold (C) = from October to December. Temperature and Relative Humidity in the proximity of the slaughter plant were recorded and are reported in Table 1. Lairage lasting was measured recording the time of loading on the truck and the time in which the first animal of the batch was stunned, and divided into two categories according to overnight lairage: Yes = batches of pigs subjected to overnight lairage (n = 17 batches, average lairage lasting 13.8 h ± 5.2); No = batches of pigs slaughtered the same day of loading on the truck (n = 55 batches, average lairage lasting 2.1 h ± 0.9).

**Table 1:** Average temperature and relative humidity [min-max] measured in the proximity of the slaughter plant.

	n. of batches	Months	Temperature	Relative Humidity
**Warm-season**	33	June to September	27.8°C [18–38°C]	58.6% [33–93%]
**Cold season**	46	October to December	11.3°C [-1-21°C]	99.0% [81–100%]

#### Animal-Based Measures (ABM)

In the present paper two types of ABM were proposed: lesions assessed on the dressing line (i.e. skin and tail lesions) and ham defects (i.e. abscesses, hematomas, muscle tear, petechial haemorrhaging, PSE and DFD-like meat, veining,).

Lesions were assessed on the body (skin lesion) and the tail (tail lesion). The same two observers (M.V. and E.S.) performed lesion assessment during the entire trial. Skin lesions were assessed on the dressing line (after the veterinary inspection point); this place was selected because it allowed to properly inspect the carcass. The tail lesions were assessed at the end of the dressing line on a raised platform, allowing to easily observe each tail. The method used for lesion score was the same proposed in the Welfare Quality^®^ [22]. Briefly, one side of the carcass was evaluated and skin lesions were considered in 4 separate areas (ear, front, middle, hind-quarters). Score was: 0 = up to 4 visible lesions; 1 = 5 to 10 visible lesions; 2 = 11 to 15 visible lesions. Tail lesions were also scored on a 0 to 2 scale (0 = absence of lesions; 1 = superficial biting along the length of the tail but no evidence of swelling; 2 = open lesion visible on the tail, the presence of scars, swelling, or partial missing of the tail). The percentage of each score was then calculated for each batch. Hence, the prevalence of lesions in each area was determined for each batch as a percentage of the sum of scores 1 and 2. Values of prevalence above 10% were considered as indicators of welfare issues. Lastly, the lesion score index (LSI), which considered both the frequency and the severity of the lesions, was then calculated in each area as follows (range 0 to 200, where 0 is absence and 200 is all animals with severe lesions [23, 24]:

LSI=(%oflesionscore1+(2*%oflesionscore2)).

**Fig 1:**
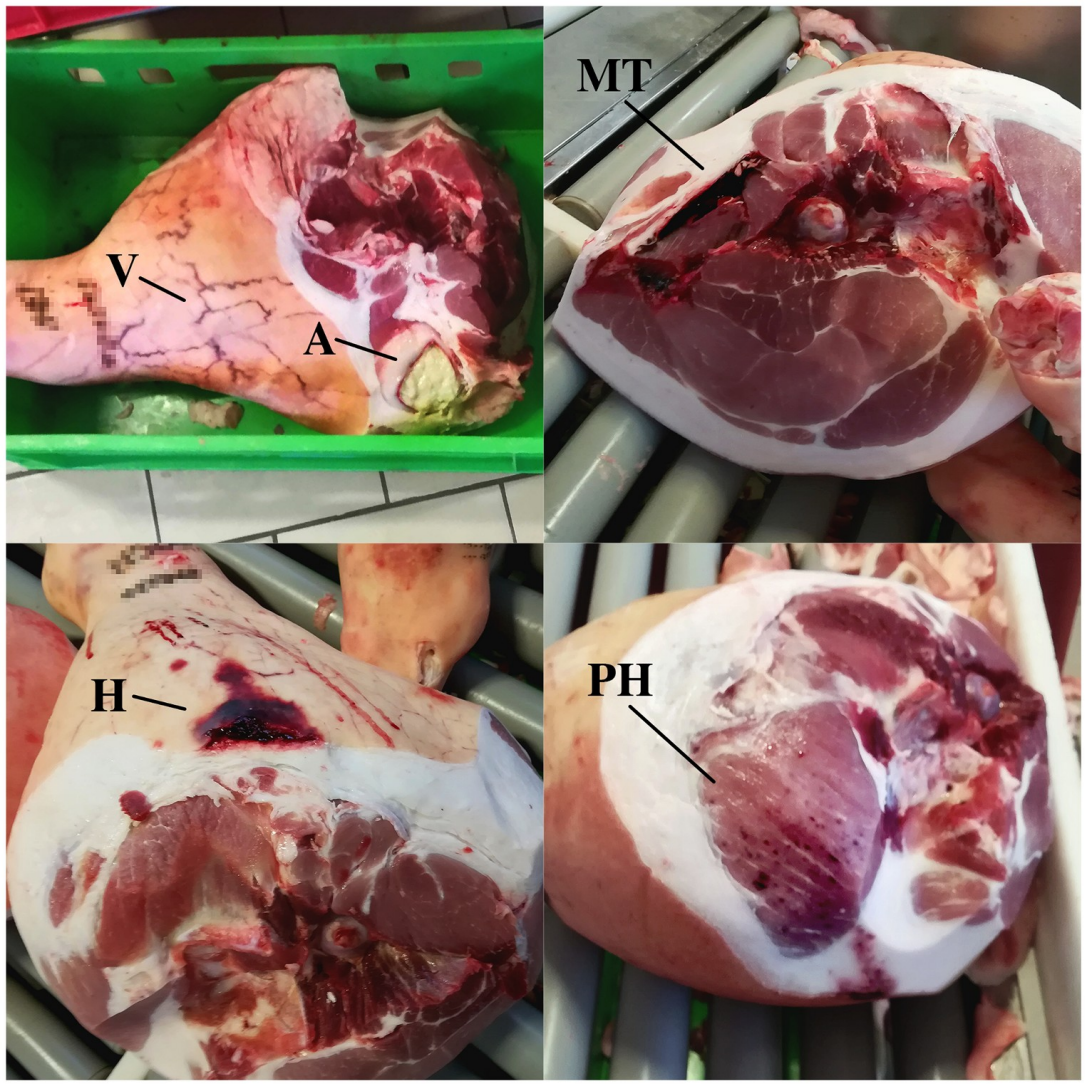
A pictorial example of the ham defects recorded in the study. V = Veining defect; A = abscess; MT = Muscle tears. H = Haematoma. PH = Petechial haemorrhaging.

The day after slaughter, all the hams of each carcass from the batch were assessed. Definition of defects was performed by a qualified technician from the slaughterhouse, officially licensed from the Parma Ham Consortium. Percentage of hams showing veining, abscessation and muscular tear were assessed according to Parma Ham disciplinary [25]. Those defects are a cause of exclusion from the PDO. Explanation of ham defects is reported in Table 2 and Fig 1.

**Table 2:** Description of the ham defects recorded in the study.

Defect	Description^a^
**PSE- like meat**	Pale, soft and exudative meat appearance.
**DFD- like meat**	Dark, firm and dry meat appearance.
**Muscle tear**	Laceration of the muscular fasciculus.
**Haematoma**	Presence of haematomas or ecchymosis under the skin that eventually extend in the muscular tissue behind and/or substantial modification of the exterior appearance.
**Abscess**	Presence of pus and a swelling area that may involve subcutaneous and/or the muscular tissue behind.
**Petechial haemorrhaging**	Areas of pericapillary bleeding in the meat.
**Veining**	Presence of evident subcutaneous venous lattice affecting the medial or entire surface of the ham.

^**a**^The description has been translated and adapted from the Parma Ham Disciplinary (CSQA[25]).

#### Carcass traits

In each batch, the carcass cold weight and lean meat percentages were recorded. Lean meat percentage was calculated using a Fat-o-Meater (FOM-SFK, Copenhagen, Denmark) and the National equation of prediction [26].

### Statistical analysis

The experimental unit was the batch since it was not possible to track each carcass and ham among the observation stages. Therefore, the average value was calculated for all the parameters.

Descriptive analysis was performed using Excel for Windows. The factorial analysis of mixed data (FAMD) was performed using the FactoRmine package in the R environment [27, 28], to assess relationships between pre-slaughter factors, skin and tail lesions and ham defects. A first explorative FAMD was conducted to observe the association between pre-slaughter factors and ABM; then a second FAMD was performed excluding the ABM not associated with pre-slaughter conditions. The principal components from this second FAMD were used to assess their relationships with carcass traits. Normality was tested for cold carcass weight and lean meat percentage and, as data were normally distributed, a LMER model (Package Lme4 [29]) was built, with carcass traits measures as dependent variables, and principal dimensions as the covariate. Farm of origin was used as a random factor, in order to consider in the model, the % of variance that the farm of origin could have on the batch characteristics.

## Results

On average, the batches were composed of 128 ± 13 pigs, raised at 83.2 ± 35.0 km from the abattoir. Lairage lasted on average 280.5 ± 330.3 minutes (corresponding to 4.7 ± 5.5 hours). A total of 43 batches were assessed in the cold season, and 33 in the warm season. The dataset is consultable at S1 File.

From the present study, overall, the batches of pigs showed severe level (i.e. prevalence above 10%) in the following welfare outcomes: haematoma in the ham (50.3% ± 26.55), tail lesions (34.8% ± 11.21), front lesions (25.24% ± 15.35), muscle tear (12.84% ± 15.49, assessed in the ham), ear lesions (11.70% ± 11.84) and petechial haemorrhaging on the ham (10.22% ± 9.41). Full results are described in Table 3.

**Table 3:** Descriptive analysis of the Animal Based Measure (ABM) observed.

ABM		mean	sd	median	min	max
***Ear lesion***						
**Ear prevalence**	%	11.70	11.84	7.00	0.00	50.00
**Ear score 0**	%	88.30	87.03	94.00	100.00	45.36
**Ear score 1**	%	6.10	6.76	3.00	0.00	31.00
**Ear score 2**	%	5.59	6.22	3.00	0.00	23.64
**Ear LSI**^1^	0–200	17.29	17.67	10.00	0.00	70.91
***Front lesion***						
**Front prevalence**	%	25.24	15.35	22.00	3.00	57.00
**Front 0**	%	74.76	82.12	80.00	99.00	26.00
**Front 1**	%	13.02	7.72	12.00	1.00	35.00
**Front 2**	%	12.22	10.16	8.00	0.00	39.00
**Front LSI**^1^	0–200	37.46	24.86	29.00	4.00	93.00
***Middle lesion***						
**Middle prevalence**	%	21.22	11.08	19.30	5.00	59.00
**Middle 0**	%	78.78	86.60	82.00	98.00	34.00
**Middle 1**	%	13.03	6.17	12.00	2.00	30.00
**Middle 2**	%	8.19	7.23	6.00	0.00	36.00
**Middle LSI**^1^	0–200	29.41	17.66	26.32	5.00	95.00
***Hind quarter lesion***						
**Hind quarter prevalence**	%	8.91	7.21	6.92	0.00	37.00
**Hind quarter 0**	%	91.09	91.40	93.00	100.00	55.00
**Hind quarter 1**	%	5.88	4.79	5.00	0.00	24.00
**Hind quarter 2**	%	3.02	3.81	2.00	0.00	21.00
**Hind quarter LSI**^1^	0–200	11.93	10.48	9.00	0.00	50.00
***Tail lesions***						
**Tail prevalence**	%	34.08	11.21	32.00	11.00	68.00
**Tail 0**	%	65.92	86.44	69.00	91.00	26.00
**Tail 1**	%	29.64	8.54	28.00	9.00	50.00
**Tail 2**	%	4.44	5.02	3.00	0.00	24.00
**Tail LSI**^1^	0–200	38.52	15.13	36.00	13.00	92.00
***Ham defects***						
**PSE-like meat**^2^	%	4.69	5.74	1.96	0.00	24.81
**DFD-like meat**^2^	%	1.02	5.97	0.00	0.00	52.78
**Haematoma**^2^	%	50.03	26.55	45.76	0.00	100.00
**Petechial Haemorrhaging**^2^	%	10.22	9.41	8.07	0.00	48.61
**Veining**^2^	%	5.46	7.26	2.63	0.00	34.32
**Abscess**^2^	%	4.06	10.55	0.00	0.00	66.67
**Muscle tear**^2^	%	12.84	15.49	7.78	0.00	96.30

^1^The values are calculated on a range of 0–200 considering the prevalence and severity of the lesions, where 0 is absence and 200 all carcasses in the batch with severe lesions.

^2^Scores were calculated as the prevalence in the batch showing the presence of the clinical sign.

### Multivariate analysis

The multivariate analysis explores the dynamic between pre-slaughter factors, and ABM measured in the carcass or the ham. The first exploratory FAMD excluded the variables which showed to contribute less than 5% on the first two dimensions and with a cos2 ≤ 0.2 (S1 File), and namely all ham defects such as PSE- and DFD- like meat, haematomas and petechial haemorrhaging. Those variables did not cluster with the others and were not likely dependent on the pre-slaughter factors assessed in this study.

**Fig 2:**
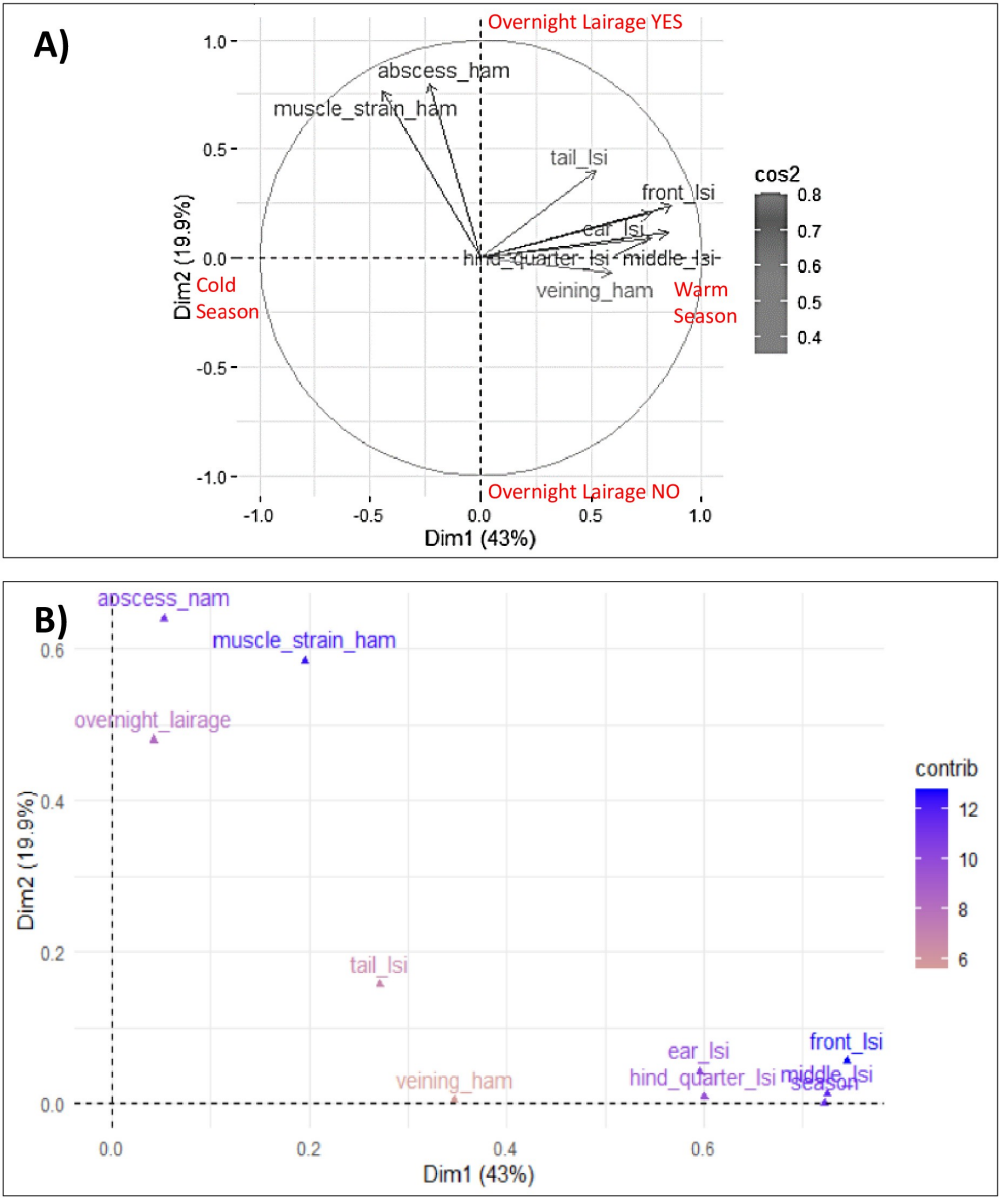
Results of FAMD analysis identifies the relationships between pre-slaughter factors (overnight lairage and season of slaughter) and animal-based measures (skin lesions, tail lesions and ham defects). A = graph of animal-based measures. Red writes represent how pre-slaughter factors are segregated in the two axes. B = all variables.

Therefore the second FAMD analysis showed that the first two dimensions accounted for 62.9% of the variability (43.0% from Dimension 1 and 19.9% from Dimension 2) (Fig 2).

Factors mostly contributing to Dim 1 were season and skin lesions (ear, front, middle, hind quarters) tail lesions and veining defect in the ham (Table 4). Factors mostly contributing to Dim 2 were lairage lasting, ham abscesses, ham muscle tears and tail lesions (Table 4).

**Table 4:** Eigenvalues, cos2 and contribute to the variables in the FAMD model. The two dimensions identified animal-based measures that clustered with the pre-slaughter factors.

		Dim.1			Dim.2		
		(Season -RF^3^)			(Overnight lairage–RF^3^)		
		eigenvalue	cos2	contribute	eigenvalue	cos2	contribute
***Pre-slaughter factors***	**Warm season**	2.29	0.96	10.56	-0.06	0.00	0.04
	**Cold season**	-1.35	0.96	6.24	0.04	0.00	0.02
	**Overnight lairage YES**	-0.82	0.12	0.77	1.87	0.65	18.96
	**Overnight lairage NO**	0.22	0.12	0.21	-0.51	0.65	5.17
***Animal-based measure***	**Ear LSI**^1^	0.77	0.60	13.87	0.21	0.04	2.13
	**Front LSI**^1^	0.86	0.75	17.34	0.24	0.06	2.82
	**Middle LSI**^1^	0.85	0.73	16.87	0.11	0.01	0.65
	**Ham LSI**^1^	0.77	0.60	13.96	0.09	0.01	0.42
	**Tail LSI**^1^	0.52	0.27	6.32	0.40	0.16	7.92
	**Veining defect in ham**^2^	0.59	0.35	8.08	-0.07	0.01	0.26
	**Ham abscess**^2^	-0.23	0.05	1.24	0.80	0.64	32.21
	**Muscle tears in ham**^2^	-0.44	0.20	4.55	0.76	0.58	29.42

^1^The values are calculated on a range of 0–200 considering the prevalence and severity of the lesions, where 0 is absence and 200 all carcasses in the batch with severe lesions.

^2^Scores were calculated as the prevalence in the batch showing the presence of the clinical sign.

^3^RF = related factors.

The position of each ABM in Fig 2A shows how they vary according to the others. If variables are in the same direction, it means that they are directly proportional. Variables in the orthogonal position are not correlated (Fig 2A). Pre-slaughter factors segregated in the two axes and Fig 2B showed how they are related to ABM. Therefore, Dim 1 was called Season related factors (-RF) and Dim 2 Overnight lairage -RF. The position of the variables in Fig 2, shows the highest LSI in skin lesions and the highest prevalence of veining defect in the batches slaughtered in summer. For the same reason, overnight lairage showed the highest frequencies of some ham defects, such as muscle tears and abscesses. Similarly, with overnight lairage, a higher tail LSI was observed.

### Association study

To evaluate the effect of Season -RF and Overnight lairage -RF on carcass traits parameters like carcass weight and lean meat percentage, an association study was performed using a linear mixed model. The results showed that those factors did not affect cold carcass weight.

When considering lean meat percentage, Season- RF was not associated with lean meat content whereas higher values on Overnight lairage -RF was related to less lean meat percentage in the carcass (P < 0.02). Full results are reported in Table 5.

**Table 5:** Association study between season-related factors (RF) and overnight lairage -RF with carcass traits parameters.

	Mean	SD	Season -RF			Overnight lairage -RF			Farm of origin	
			Estimate	Chi-sq	*P-value*	Estimate	Chi-sq	*P-value*	% of variance	sd
**Cold carcass weight (kg)**	139.73	6.52	-0.41	1.25	0.26	-0.39	0.77	0.38	21.46	4.63
**Lean meat (%)**	51.26	1.29	0.04	0.31	0.58	-0.17	5.23	0.02	1.52	1.23

## Discussion

The results evidenced that the batches of pigs assessed in the present study had main welfare issues (average prevalence above 10%) of skin lesions (especially in the front and middle area of the body), tail lesions and, in the hams, haematomas, muscle tears and petechial haemorrhaging.

This could reflect the presence of negative behaviour in the pre-slaughter phases [6]. Skin lesions are mainly the results of fighting and mounting behaviour, which principally occurs when unfamiliar pigs are mixed at the farm, during transport and lairage [8]. Loin bruises and claw lesions in the middle area can arise from overcrowding and high stocking density, or inappropriate pig handling [30]. Muscle tears in the ham and haematomas in addition can derive from many risk factors, in which the most common are inappropriate handling (e.g. loading and unloading), fighting after mixing unfamiliar pigs, inadequate floor conditions that can facilitate slipping, falling, or muscular fatigue [30]. Tail lesions are considered an iceberg indicator and it can occur during all the phases of pig production. Tail lesions are often deep open lesions that can involve also muscles and bones, and that in the worse scenario can result in a complete loss of the tail. Therefore this type of lesion takes time to heal, and the presence of the scar can be related to previous farm conditions (e.g. in the post-weaning phase) and not only to the pre-slaughtering phase [17].

The use of multivariate analysis is considered one of the leading methods to identify association among factors that seems not related and to avoid misinterpretation derived by the collinearity among variables [8]. The results enlighten two main dimensions: the first was constituted by variables related to the season of slaughter, and the second by variables related to overnight lairage. The season of slaughter was associated with the frequencies of skin lesions, tail lesions and veining defect in the ham. In particular, the stronger effect was observed in all the areas of skin lesion (ear, front, middle and hind quarter), while a slighter contribution was observed in tail lesions. In the present studies, those ABM showed an increased prevalence in summer. The literature reported different results on the seasonal effect of skin lesion and ham defects in pigs. The present results are in contrast with what observed by Dalla Costa et al [31] and Cobanovic [32], who observed increased skin lesions in winter, and Arduini et al [20], who reported increased muscle tears in the cold seasons (winter and autumn). The results of the present study agreed with what has been claimed by Bottaccini et al. [17] and Goumon et al [33]. In the present study, the behaviour was not assessed, while Gourmon et al [33] reported that pigs in the cold season are more reluctant to move and spent more time lying close to each other, avoiding fighting. Heat stress, on the contrary, has been found to interact with social stress in pigs in the modulation of the immune response [34], showing that when pigs were heat-stressed, the relationships between social behaviour and leukocyte numbers became more pronounced. Therefore, according to the same study, under high temperatures, a relationship between high neutrophils and low neutrophils: lymphocytes ratio with a high level of aggressiveness has been observed [34]. This requires further investigation exploring the effect of season on pre-slaughter aggressiveness and skin lesion. Besides, veining defect showed a higher occurrence during the warm season.

The effect of season on veining defect has been inconsistent among studies. In fact, veining defect has been observed more frequently in autumn [20] or spring [17]. This difference is imputable to the scarce knowledge behind the physiology underlying the veining defect. Overall, the season has confirmed to impact ABM, showing differences between studies. Those discrepancies should be ascribable to differences in the slaughter plant management, in particular, the temperature and humidity in the year of the assessment, countries and categories of pigs. Especially with regards to slaughter management, more efforts should be put into recording data showing seasonal variation in the slaughterhouse practices. For example, it has been reported that some slaughterhouse changes the temperature of the scalding water in summer and that it has been linked to the prevalence of veining defect [17]. Also, the stockman can play a role in the development of skin lesion due to inappropriate handling: an increase of injuries and skin lesions has been reported with an undertrained or insufficient number of employees [30], a condition that may occur in some periods due to training of new employees or following the highest market request of meat products (e.g. during festivities).

Overnight lairage was related to ham defects such as abscess and muscle tears, as well as the increase of tail lesions. To the best of the Authors’ knowledge, no literature was found exploring the effect of overnight lairage on the inflammation processes or the prevalence of muscle tears It could be hypothesized that long lairage lasting might worsen previous infectious and/or traumatic conditions, such as abscesses and muscle tears. This result is innovative, but it requires further studies to be better explained. These batches showed also an increased score in tail lesions. The contribution of tail lesion score in lairage -RF seems to be consistent with the knowledge that pigs subjected to overnight lairage undergone stressful conditions [35–37].

The two dimensions identified from the multivariate analysis, corresponding to pre-slaughter -RF, showed no association with cold carcass weight. Lairage -RF instead, were inversely related to lean meat content. In the study of Murray et al [38], it has been reported that a lairage of 0–5 h did not affect meat quality. In the present study, overnight lairage could last up to 20 hours, and therefore the effect on pig metabolism can be different. Moreover, if lairage itself is considered one of the main factors affecting meat quality [35], it is well known that all the ABM included in this dimension (i.e. ham abscesses, muscle tears and tail biting) are a long-term source of stress, pain, and inflammatory conditions in pigs. These conditions can alter pig physiology [39]. The activation of the sympathetic nervous system in pigs undergoing pre-slaughter stress has been found responsible for the release of cortisol from the zona fasciculate of the adrenal cortex [39, 40]. A study from Foury et al. [41], found that carcass fat content was higher and estimated carcass lean meat content was lower with increasing urinary levels of cortisol and adrenaline, enhancing the accumulation of fat at the expense of muscle proteins. Besides, the activation of the immune system through the involvement of cytokine, chemokines, and leukocyte, following lesions and injuries has been found in carcasses also after slaughter [8, 10]. Moreover, the immune system is known to modulate the muscular metabolism, especially with regards to protein and fat content, and therefore it is reasonable to hypothesize differences in the lean meat percentage observed at slaughter in batches with a higher prevalence of ham abscesses or muscle tears. Besides, recent research [42] has been proposed that immune activation, especially the involvement of cytokines compounds, could be a major factor in social interaction in pigs, as an example, in the development of tail biting and tail lesion outbreak. For this reason, one limitation of the study is the absence of physiological indicators that could have been used to validate the present hypotheses. The present results pose new questions on the pain and distress of pigs submitted to overnight lairage lasting, and on how it might affect physiology and meat quality, that should be addressed in further studies.

Some ABM resulted not associated with pre-slaughter factors nor other ABM, therefore they were excluded from further analysis in the present study. It does not mean that these ABM, which have shown also high prevalence (haematomas and petechial haemorrhaging in the ham), have not a strong impact on animal welfare, but it highlights that they were probably depending on the different pre-slaughter risk factor, actually not measured in the present study (e.g. transport or stunning method efficacy). Further studies with a more comprehensive assessment of pre-slaughter factors, will allow important insights on welfare assessment at slaughter.

Further research is deemed needed. The use of ABM from ham assessment showed promising application since they were not correlated to skin and tail lesions, therefore they might be useful to get a more comprehensive and complete retrospective welfare assessment in pigs.

## Conclusion

The study found that in the batches observed, the main welfare issues were skin lesions, ham haematomas, tail lesions, ham tear and petechial haemorrhaging, which let to hypothesize pre-slaughter issues in negative social behaviour and pig handling. The multivariate analysis reported the association between skin lesions, tail lesions, veining ham defect and the season of slaughter, with all the ABM higher in the warm season. Another association was found between overnight lairage with ham tears, ham abscesses and tail lesions. Overnight lairage, ham abscesses and ham muscle tears were then found to be associated with lean meat percentage, sustaining the need for a further study addressing the physiological changes caused by stress and immunity impairment and their effect on carcass traits. The use of ham defects as ABM seems promising since they were not correlated to skin lesions, therefore they can provide new insight on the retrospective welfare assessment at the slaughter plant.

## Supporting information

S1 File Complete dataset used for the statistical analysis. (XLSX)

S2 File Eigenvalues, cos2 and contribute to the variables in a first explorative FAMD model. **The two dimensions identified animal-based measures that clustered with the pre-slaughter factors**. Table A: Percentage of variance explained by each dimensions. Table B: Eigenvalues, cos2 and contribute of the variables. Table C: Eigenvalues, cos2 and contribute of each batch. Graphs: graphs of variables.
(XLSX)
